# The Trade-Off between Optimizing Flight Patterns and Human Health: A Case Study of Aircraft Noise in Queens, NY, USA

**DOI:** 10.3390/ijerph15081753

**Published:** 2018-08-15

**Authors:** Zafar Zafari, Boshen Jiao, Brian Will, Shukai Li, Peter Alexander Muennig

**Affiliations:** 1 Global Research Analytics for Population Health, Mailman School of Public Health, Columbia University, New York, NY 10032, USA; jiaoboshen@gmail.com (B.J.); lishukai@pku.edu.cn (S.L.); pm124@cumc.columbia.edu (P.A.M.); 2 Pharmaceutical Health Services Research, University of Maryland School of Pharmacy, Baltimore, MD 21201, USA; 3 Queens Quiet Skies, P.O. Box 604888, Bayside, New York, NY 11360-4888, USA; brian.f.will@gmail.com

**Keywords:** cost-effectiveness, aircraft noise, cardiovascular disease, Markov model

## Abstract

*Objectives:* Airports in the U.S. have gradually been transitioning to automated flight systems. These systems generate new flight paths over populated areas. While they can improve flight efficiency, the increased noise associated with these novel flight patterns potentially pose serious health threats to the overflown communities. In this case study, we estimated the monetary benefits relative to health losses associated with one significant change in flight patterns at LaGuardia Airport, year-round use of “TNNIS Climb”, which happened in 2012 as a result of flight automation in New York City. Prior to that, the use of the TNNIS Climb was limited to the U.S. Open tennis matches. *Methods:* We developed a decision-analytic model using Markov health states to compare the costs and quality-adjusted life years (QALYs) gained associated with the limited use of TNNIS (old status quo) and the year-round use of TNNIS (current status quo). The TNNIS Climb increases airplane noise to above 60 decibels (dB) over some of the most densely populated areas of the city. We used this increased exposure to noise as the basis for estimating ground-level health using data from sound monitors. The total costs (including both direct and indirect costs), QALYs, and the incremental cost-effectiveness ratio (ICER) were estimated for the limited versus the year-round use of the TNNIS Climb. *Results:* The incremental lifetime costs and QALYs per person exposed to noise associated with the limited versus the year-round use of TNNIS was $11,288, and 1.13, respectively. Therefore, the limited use of TNNIS had an ICER of $10,006/QALY gained relative to the year-round of TNNIS. Our analyses were robust to changes in assumptions and data inputs. *Conclusions:* Despite increases in efficiency, flight automation systems without a careful assessment of noise might generate flight paths over densely populated areas and cause serious health conditions for the overflown communities.

## 1. Introduction

Despite a vast global airspace, aircrafts have historically tended to follow a predetermined pattern that is somewhat akin to roads for automobiles [1,2]. For a long time, flights were primarily guided by radar and radio [2]. As with other areas of transportation, however, automated flight systems are gradually being rolled out. NextGen is one these automated systems that uses GPS, data from other flights, and atmospheric conditions to optimize flight patterns [1,2,3]. NextGen has also the potential to reduce pollution, flight time, costs, and accidents due to human error [4]. Its use, however, has resulted in changes in flight patterns that might go over densely populated residential areas, which pose public health threats by potentially exposing people on ground to a loud and continuous noise [1,2]. One example of the impact of NextGen can be found in New York City, where it effectively led to the year-round use of the otherwise restricted “TNNIS Climb” at LaGuardia Airport (LGA) since its implementation.

The TNNIS Climb had been historically used only during the U.S. Open, a large tennis tournament held within a park in Queens, NY, USA, that is adjacent to LGA airport. Historically, flights departing from LGA flew over the tennis stadium in Flushing Meadows and over other sparsely populated areas, such as the East River. However, because the roar of jet engines was loud enough to disrupt the matches, flights were instead diverted over densely populated residential neighborhoods of Queens (mainly Community Boards 7 and 11), NY, USA, during tennis matches. This flight, which uses runway 13 at LGA, is coded TNNIS and is known as the TNNIS Climb [5,6,7].

In the era of NextGen, use of runway 13 at LGA has become a common route of departure, and TNNIS Climb has become year-round [6,8]. The trajectories of the TNNIS and the Whitestone Climbs which are the two major departure routes of the runway 13 at LGA are provided elsewhere [9,10]. The year-round use of TNNIS has produced large increases in aircraft noise for residential areas of Community Boards 7 and 11 of Queens, NY, USA. These neighborhoods were previously quiet. High levels of exposure to aircraft noise has been linked to development of serious physical and mental health conditions such as cardiovascular disease (CVD) and anxiety [11,12,13,14,15]. In this paper, we quantified the potential health and economic consequences of the year-round use of TNNIS at LGA in Queens, NY, USA, and asked whether its benefits would outweigh the noise-associated health risks experienced by people in what were once quiet communities.

## 2. Materials and Methods

We developed a simulation model to evaluate the cost-effectiveness of limiting the year-round use of TNNIS in Queens, NY, USA. For this purpose, we quantified the lifetime health and economic outcomes for the two comparison arms of our study: (1) the limited use of TNNIS (old status quo); and (2) the year-round use of TNNIS (current status quo). We measured health outcomes in terms of the quality-adjusted life years (QALYs). Our study adopts a societal perspective, and thus, we modelled both direct and indirect (productivity loss) medical costs in our main analyses. We finally measured the incremental costs over the incremental QALYs to calculate the incremental cost-effectiveness ratio (ICER) of the limited vs. the year-round use of TNNIS in Queens, NY, USA.

### 2.1. Impacts of the Year-Round Use of TNNIS on Noise

The Port Authority of New York and New Jersey measured the average day and night levels (DNL) of noise [16]. They specified noise contours over the affected communities of Queens, outlining which areas were exposed to 55+, 60+, 65+, 70+, and 75+ decibels (dB) DNL [16]. Noise corridor data showed that all year use of TNNIS has produced loud and frequent noise for people on ground, especially persons living in the Community Boards 7 and 11 of Queens, NY, USA [5,6,7,16]. The Port Authority of New York and New Jersey has created a real-time online noise tracker, through which we verified various time point data in the existing noise corridor under the TNNIS Climb. These levels spike during aircraft movements in Queens, NY, USA, within the Community Boards 7 and 11 [5]. This online noise tracker demonstrates that noise levels from nearby monitors can rise from below 55 dB to above 90 dB when an aircraft overflies sound monitors on ground [5]. While data from the online noise tracker and noise estimates from the Port Authority of New York and New Jersey [5,16] might suggest a larger impact, we assumed that people living within the 60 dB DNL noise contour would have been exposed to noise below 50 dB had not TNNIS become year-round.

### 2.2. Health Impacts of the Year-Round Use of TNNIS

The European’s Environmental Noise Directive has recommended 55 dB as the threshold noise level for day, evening, and night (L_den_) periods [11,17,18], whereas in the U.S. the allowable limit is 65 dB DNL [19]. A difference of 10 dB in noise makes a 65 dB ten times the intensity (power) and two times the loudness of a 55 dB. Multiple studies have shown worst health conditions associated with noise exposure above a 55 dB threshold [14,20,21]. While there is evidence of impacts on health also at lower levels [22], in this study we conservatively assumed that 60 dB DNL threshold is the line above which health problems arise. In doing so, we ensure that we: (1) are not underestimating the health impacts, (2) are not overestimating the number of people who are impacted by the noise, and (3) are able to line up our model inputs around more uniformly available metrics. The downside of this approach is that we are neither exploring European standards nor U.S. standards for aircraft noise exposure.

To identify the health impacts of aircraft noise after the year-round use TNNIS, we estimated the number of persons in the Community Boards 7 and 11 of Queens, NY, who were living within the 60 dB DNL noise contour. The details of our estimation can be found in the Supplementary Material (Figure S1).

Several studies have quantified how transport activities impact on ground [23,24], as well as the potential deleterious effects of aircraft noise on both physical and mental health [25,26,27,28,29]. In this study, we modeled the increased risk of cardiovascular diseases (CVD) (ICD-10 Chapter I) and generalized anxiety disorder associated with aircraft noise [11,12,13,14,15]. Because it interferes with sleep, aircraft noise plausibly has much broader effects on health, economic productivity, and educational outcomes among children [11,17,18,25]. However, since we did not have adequate data, in this study we only modeled CVD and anxiety.

We derived the relative risks of CVD (ICD-10 Chapter I) and generalized anxiety disorder associated with aircraft noise from the literature [14,15]. Hansell et al. measured the relative risk of CVD (ICD-10 Chapter I) associated with daytime exposure to noise as 1.14 (for >60 vs. <50 dB) [14]. In addition, Hardoy et al. showed that the aircraft noise is associated with generalized anxiety disorder with an odds ratio of 2 [15]. While they did not further separate out the risk of anxiety associated with different levels of noise, we assumed that their estimated odds ratio would be applicable to our dichotomized noise exposure levels (>60 vs. <50 dB DNL). We then converted this odds ratio to relative risk, value of which can be found in Table 1.

### 2.3. Economic Impacts of the Year-Round Use of TNNIS

We analyzed the Bureau of Transportation Statistics records [42] to explore the mean impact of the year-round use of TNNIS, which began in 2012. We sought evidence of changes in utilization and delays from LGA and John Fitzgerald Kenney (JFK) airport. We included JFK because there is conflict between the airspace of LGA and JFK. Therefore, optimizing flights at LGA could improve performance at JFK.

For domestic flights at LGA, the Bureau of Transportation Statistics shows that the average on-time departures dropped from 82% in 2012 to 77% in 2016. Likewise, on-time arrivals declined from 77% in 2012 to 72% in 2016 [42]. Based on the 2016 Air Traffic Report from the Port Authority of New York and New Jersey [43], the total number of domestic and international flights at LGA and JFK has not been changed from levels seen in 2007 [43]. Neither these documents nor documents obtained under the Freedom of Information Act (FOIA) request provided to us explained why delays have increased even as the number of flights has remained constant.

While on-time status and utilization is a part of the public record [42], data on flight times are not. A letter from the Global Gateway Alliance, an advocacy group, indicated that TNNIS reduced flight delays and fuel consumption at JFK airport [44]. They found that, during a 5-day TNNIS Climb test period in February 2012, the average delay time at JFK dropped from 45.7 to 25.3 min [44]. However, they did not provide data or methods. Nor did they provide any information on real-world, long-term impacts after implementation of NextGen. Given that real world data do not show evidence of any improvement in utilization or delays, we used this data as a thought experiment that represents the ‘best-case’ scenario for the TNNIS Climb. We assumed all delays happen in the sky during fuel burn. We modeled costs associated with flight delays through two components: (1) operating costs (including fuel, labor, and other costs); and (2) productivity losses among passengers using the average number of passengers per flight.

(1) Operating costs

We calculated operating costs via the following formula:

(1) $$\;C_{i}=DELAY_{i}\times SPEED\times CASM\times SEAT\times FLIGHT_{i},\;$$

where *i* represents the limited vs. the year-round use of TNNIS. *DELAY*, *SPEED*, *CASM*, *SEAT*, and *FLIGHT* represents delay time, average speed of an aircraft, costs per available seat mile (including fuel, labor, and other costs), average number of seats per flight, and average number of delayed flights per year, respectively.

We used an average delay time of 45.7 min per delayed flight for the time that the use of TNNIS was limited, and 25.3 min after the year-round use of TNNIS from a report [44]. The same report also claimed that making TNNIS year-round reduced the number of delayed flights from 204 to 12 over a 5-day test period [44]. We multiplied these numbers by 73 to obtain the annual number of delayed flights before and after the year-round use of TNNIS. While we did not find data to represent the distribution of delays in different phases of a flight (i.e., take-off, climb, cruise, descent, and landing), we assumed that such delays would happen during the cruise phase for the following two reasons: (1) the cruise phase takes up the majority of a flight’s time; and (2) the assumption captures the maximum potential savings in operating costs in favor of the year-round use of TNNIS because of the higher average aircraft speed during the cruise phase compared to the climb and descent phases. The average cruise speed of an aircraft was assumed to be 900 km/h [45]. The average costs per available seat mile, and average number of seats per flight was estimated to be 11.6 cents [46], and 176 [47], respectively. The incremental annual operating costs per person exposed to noise for the limited relative to the year-round use of TNNIS are presented in Table 1.

(2) Productivity losses

In addition, we calculated productivity losses using the following formula:

(2) $$P_{i}=DELAY_{i}\times WAGE\times SEAT\times FLIGHT_{i},$$

where *i* represents the limited vs. the year-round use of TNNIS, and *WAGE* represents the average salary for a traveler. We got the income distribution for airline travelers across the following four income groups: <25 K, 25 K to <50 K, 50 K to <100 K, and 100 K and above [48], and multiplied it by the estimated mean income for each group derived from the U.S. Census Bureau [49]. The rest of the parameters were the same as described earlier. The incremental annual productivity loss per person exposed to noise for the limited relative to the year-round use of TNNIS is presented in Table 1.

### 2.4. Simulation Model

We developed a probabilistic Markov model based on CVD and anxiety as the two relevant health effects. Pre-existing CVD has been associated with almost two-fold increase in the risk of a subsequent CVD [14,15,16,17,18]. We therefore based our Markov model on three health states including “no prior history of CVD”, “prior history of CVD”, and “death”. We also modeled onset of the generalized anxiety disorder as an event in our analysis. The starting age of the cohort was chosen as 41 to be a representative of our target population in Queens, NY, USA [50]. Every cohort was followed over a lifetime using a Markov cycle length of one year.

### 2.5. Model Parameters

#### 2.5.1. Background Rates and Probabilities

The background and CVD mortality rates were derived from the U.S. Life Tables [31] and a 2016 updated report from the American Heart Association [32]. In our model, participants were assigned a background risk of developing CVD and anxiety [32,34]. In addition, increased risk of CVD and anxiety for people with pre-existing CVD were modeled from related studies [33,35] (Table 1).

#### 2.5.2. Other Costs Parameters

The direct medical costs of CVD were drawn from a study by Nichols et al. [33]. Previous studies reported on the ratio of indirect to direct costs of CVD [37,51]. We used this ratio to calculate the indirect medical costs of CVD. In addition, direct and indirect medical costs of anxiety were derived from a study by Greenberg [36]. All costs were adjusted to 2016 U.S. dollar (Table 1).

#### 2.5.3. Health State Utility Values

Health utilities are scaled from 0 to 1, with 0 equals to death and 1 equals to perfect health. We obtained values for CVD from literature [38,39]. To estimate the health utility of anxiety, we first derived the measures for the eight subscales (i.e., bodily pain, general health, mental health, physical functioning, role limitations due to emotional problems, role limitations due to physical problems, social functioning, and vitality) of the SF-36 (Medical Outcomes Study Short-Form 36-Item Health Survey) that were used to derive an estimate for the health-related quality of life associated with “no to minimal anxiety” by Brenes [52]. We then converted these values to the EuroQol-5D (EQ-5D) overall score using a validated mapping algorithm by Ara and Brazier [53]. When both CVD and anxiety happened in the same cycle, to avoid their overlapped impact on utility, we only modeled CVD-related disutility. All the health state utility values are presented in Table 1.

#### 2.5.4. Analysis

We ran our model based upon the base-case values for parameters (Table 1). We calculated total direct and indirect costs as well as changes in QALYs associated with the year-round use of TNNIS as well as its limited use. All future costs and health outcomes were discounted at 3%.

To compute uncertainty across all variables in the model, we performed a probabilistic Monte Carlo simulation with 10,000 random draws from the probability distributions of model parameters (Table 1). We presented our results both in terms of a cost-effectiveness plane and a cost-effectiveness acceptability curve. The latter indicates the probability of cost-effectiveness of the limited vs. the year-round use of TNNIS at different willingness-to-pay (WTP) values. WTP is the maximum price that policy makers are willing to pay in order to gain one QALY [54]. Our simulation model was constructed in TreeAge Pro 2017 (TreeAge Software Inc., Williamstown, MA, USA) [55].

In addition, to evaluate the robustness of the model outcomes against changes in core parameters including all direct medical costs, productivity losses, all rates and probabilities, utility values, and discount rate, we performed a large number of one-way sensitivity analyses.

Finally, because the allowable noise limit in the U.S. is 65 dB DNL [19], we conducted a scenario analysis in which we limited our target population to those exposed to noise above 65 dB DNL after the year-round use of TNNIS. In this scenario, we assumed that health conditions associated with noise would only occur upon exposure to noise above 65 dB DNL. Details of our estimation for number of people living within the 65 dB DNL noise contour can be found in the Supplementary Material (Figure S2). We investigated the cost-effectiveness outcomes of the limited vs. the year-round of TNNIS in this scenario analysis.

## 3. Results

### 3.1. Base Case Results

Table 2 presents the expected lifetime model outcomes per person exposed to noise for both the limited and the year-round use of TNNIS arms. We showed that the limited use of TNNIS was associated with more societal costs and QALYs compared to its year-round use. On average the incremental lifetime costs, and QALYs associated with the limited use of TNNIS, for an average person exposed to noise, was estimated to be $11,288, and 1.13, respectively. This resulted in an ICER of $10,006/QALY. Therefore, despite possible benefits of the year-round of TNNIS in terms of decreasing operating costs and productivity losses, the ICER of the limited vs. the year-round use of TNNIS fell below $50,000/QALY as a commonly used threshold for the cost-effectiveness analysis [56].

### 3.2. Probabilistic Results

**Figure 1:**
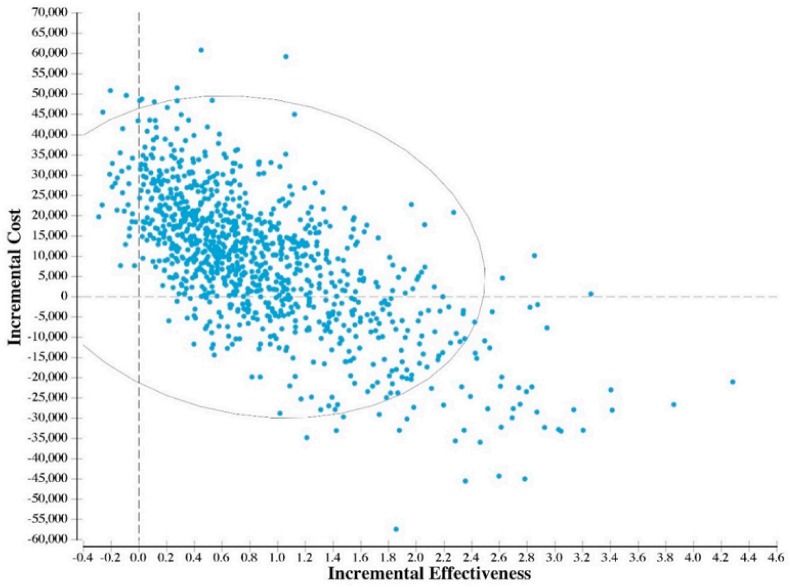
Cost-effectiveness plane for the limited vs. the year-round use of TNNIS Climb at La Guardia Airport (LGA) in New York City.

Figure 1 indicates the results of the probabilistic analyses in a cost-effectiveness plane. In 25% of the simulation runs, the limited use of the TNNIS Climb seen before 2012 was shown to be cost-saving, meaning that it would save both money and health. In addition, in 60% of the simulation runs, the ICER of the limited vs. the year-round use of TNNIS fell below $100,000/QALY.

**Figure 2:**
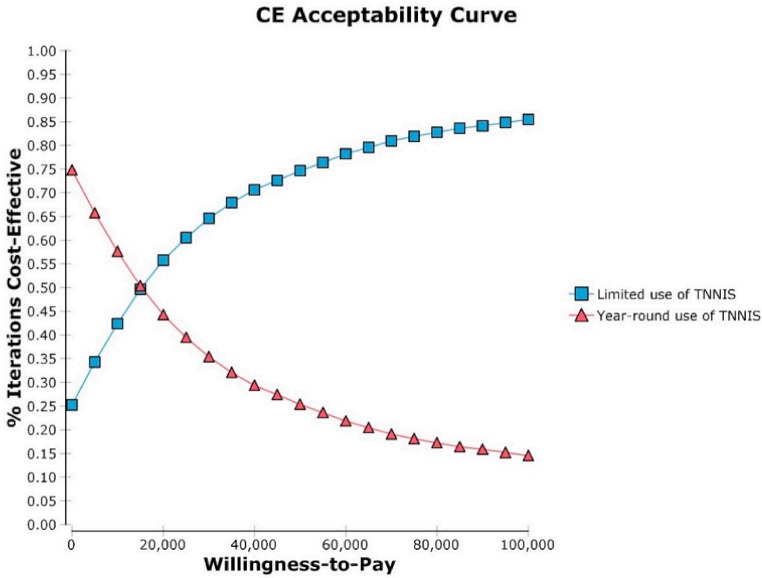
Cost-effectiveness (CE) acceptability curve for the limited vs. the year-round use of TNNIS Climb at La Guardia Airport (LGA) in New York City.

Figure 2 represents the results of the cost-effectiveness acceptability curve. At the WTP threshold value of $0/QALY, there was a 25% chance that the limited use of TNNIS would become cost-effective relative to the year-round use of TNNIS. This probability was estimated to be 75%, and 85%, at the WTP threshold value of $50,000/QALY, and $100,000/QALY, respectively.

### 3.3. One-Way Sensitivity Analysis

One-way sensitivity analyses indicate the robustness of the overall ICER in response to changes in core input parameters of the model. The most influential input parameter of our model was the relative rate of anxiety associated with aircraft noise. If there were no statistical association between anxiety and noise (relative rate = 1), the ICER of the limited vs. the year-round use of TNNIS would be increased to $208,107/QALY. In contrast, if the relative rate of anxiety due to noise exposure (>60 vs. <50 dB DNL) were increased to 3.06, the limited use of TNNIS would become a cost-saving option compared to the year-round use of TNNIS. The impact of changes in other input parameters of our sensitivity analyses was small and the ICER of the limited versus the year-round use of TNNIS remained below $100,000/QALY. The details of these analyses can be found in Table 3.

### 3.4. Scenario Analysis

We ran a scenario in which we increased the noise threshold above which health conditions arise from 60 dB to 65 dB. Accordingly, we estimated the number of persons of Community Boards 7 and 11 of Queens, NY, who were living within the 65 dB DNL noise contour due to the year-round use of TNNIS. Under this scenario, the limited use of the TNNIS Climb was associated with additional $78,288 costs and 1.13 QALYs, which resulted in an ICER of $69,401/QALY gained that still falls below the recommended 50K or 100K thresholds for cost-effectiveness [54].

## 4. Conclusions

Flight pattern optimization can, in theory, produce profound societal benefits. These include reduced atmospheric pollution, gains in productivity from reduced flight time, and increases in the timely delivery of products and services. NextGen is a recently implemented automated system at airports that optimizes flight patterns in the sky and improves flight efficiency. Airports are one of the primary points of commercial activities, and optimizing flight patterns can have positive spillover effects on the economy. However, it can also have negative impacts on health, resulting in unintentional harm and unforeseen costs. This, in turn, also produces negative spillover effects. 

In this study, we examined the potential health and economic impacts of a change in one flight route, TNNIS Climb, in NY. This route was originally used only during U.S. Open tennis tournament, but has become year-round since NextGen was implemented in 2012. We found that the ICER of the limited use of TNNIS (old status quo) would be $10,006/QALY gained compared to its year-round use (current status quo). This suggests that, based on a subset of health and economic endpoints that was modelled in this study, it is likely that limiting the use of TNNIS would be cost-effective relative to its year-round use. Doing so would prevent much more disease at a much lower cost than commonly used clinical health prevention modalities, such as colon cancer screening or mammography [54]. Because we found that limiting the use of TNNIS would result in an ICER below the recommended WTP threshold values for cost-effectiveness [56], we did not attempt to add further cost savings from productivity or other spillover effects for people on ground, such as future lost tax revenue, social service consumption, and crime costs associated with lower educational attainment [57]. Future studies that focus on less densely populated sound corridors with smaller airports may show fewer health costs, and may therefore need to account for some of these other important health and economic endpoints. 

This provides a segue into the limitations of our study. First, our study focuses on the increased use of one flight route that might have been influenced by the implementation of NextGen in one location. It does not speak about the broader trade-offs produced by NextGen in other locations as we deem critically important to explore impacts locally on a case-by-case basis. Therefore, our model is available on demand to the research community so that it can be modified for nearly any local context. Second, it is challenging to estimate the exact noise exposure associated with overflights on ground. We used sound corridor data from the Port Authority and real-time sound data. While the real-time observations of sound monitors show sounds in excess of 90 dB when an aircraft overflies the residential areas of Community Boards 7 and 11 of Queens, NY, we did not have continuous sound monitor data. We assumed that the noise exposure would have dropped to levels around or below the baseline 50 dB for people living within the 60 dB DNL noise contour had not TNNIS been used year-round. Also, the real-time system we used shows the size of the aircraft, but generally does not show all aircrafts in the area. Third, we only modeled CVD and anxiety as health consequences of noise even though there are a wider array of potential health and economic endpoints of aircraft noise. Our model is similar to a previously published cost-effectiveness model exploring the health impacts of aircraft noise [58], and we were not able to incorporate other studies of noise and health [26,27,28,29]. This is because the use of other studies would require that we make broader assumptions surrounding the dose-response relationship between noise and other types of health endpoints. As such, our results solely rely on the applicability and generalizability of the previous findings by Hansell et al. [14], in association of noise and CVD, and Hardoy et al. [15], in association of noise and anxiety, to the setting of our study. Fourth, Hardoy et al. provided an overall estimation for risk of anxiety due to aircraft noise with no specification for noise levels [15]. In this study, we assumed that the relative risk of developing anxiety as reported by Hardoy et al. would be applicable to our dichotomized noise exposure levels (>60 vs. <50 dB DNL), and that we assumed noise below 50 dB DNL would not be harmful for health. This has some support from literature, as studies have shown 55 dB DNL is the threshold at which health problems would arise [11,14,17,18,20,21]. To account for such uncertainty, in our sensitivity analyses, we used a wide range of error in our measure of association of anxiety and the noise categories of our model. Finally, efficiencies are associated with fewer emissions and air pollution. However, there is little information on how varying flight patterns over urban areas impacts particulate concentration on ground, and this potentially important benefit of the year-round use of TNNIS was not included.

Our study explores the cost-effectiveness of one air route in one city that insists over an unusually densely populated area. Though our estimates of health impacts were informed from published studies, it is very difficult to fully account for the broader economic impacts of the year-round use of TNNIS. Our study should by no means be taken as a blanket assessment of changes to flight patterns that might reduce airline fuel consumption, increase productivity, and reduce global warming. However, they point to the strong need for careful study of public health impacts of such changes before they are implemented. NextGen holds great potential for improving our lives. However, it also appears to produce an increase in disability and death, at least in New York City. Most people have some experience with unpleasant noise in their environment (be it sirens, honking, or aircraft), yet remarkably little is known about it or is done about it. We hope that models such as ours can be used to better understand the trade-offs that new technologies bring.

## Figures and Tables

**Table 1:** Parameters used for the Markov model to evaluate the cost-effectiveness of the limited vs. the year-round use of TNNIS Climb at La Guardia Airport in New York City.

Parameters	Mean (Standard Error/Range)	Distribution	References
**Probabilities**			
Population of Queens affected by TNNIS noise	83,807	-	[16,30]
Background mortality rates	U.S. Life Tables		[31]
Risk of developing a CVD event (by age)			[32]
35–44	0.0015 (0.0004)	Beta	
45–54	0.0071 (0.0018)	Beta	
55–64	0.0149 (0.0037)	Beta	
65–74	0.0266 (0.0067)	Beta	
75–84	0.0478 (0.0120)	Beta	
85+	0.0681 (0.0170)	Beta	
Relative risk of CVD among those with prior history of CVD	1.965 (1.67–2.30)	Log-normal	[33]
Risk of an anxiety disorder	0.18 (0.0070)	Beta	[34]
Relative risk of anxiety for CVD patients	1.66 (1.49–1.82)	Log-normal	[35]
Relative risk of CVD for aircraft noise exposure	1.14 (1.08–1.20)	Log-normal	[14]
Relative risk of anxiety for aircraft noise exposure	1.79 (1.00–3.06)	Log-normal	[15]
**Costs**			
Direct medical costs of CVD	23,229 (5807)	Gamma	[33]
Direct medical costs of anxiety	2814 (704)	Gamma	[36]
Indirect medical costs of CVD	12,837 (3209)	Gamma	[33,37]
Indirect medical costs of anxiety	313 (78)	Gamma	[36]
Total incremental operating costs (limited vs. year-round use of TNNIS)	127,040,655 (31,760,164)		Author estimation
Incremental operating costs per person exposed to noise (limited vs. year-round use of TNNIS) (Total divided by 83,807)	1516 (379)	Gamma	
Total incremental productivity loss (limited vs. year-round use of TNNIS)	$19,956,042 ($4,989,010)		Author estimation
Incremental productivity loss per person exposed to noise (limited vs. year-round use of TNNIS) (Total divided by 83,807)	238 (59)	Gamma	
**Utilities**			
Disutility associated with a CVD event	0.283 (0.0130)	Beta	[38,39]
Disutility associated with anxiety	0.16 (0.04 *)	Beta	[40]
Utility of prior history of CVD event health state	0.844 (0.0096)	Beta	[38,39]

All costs are adjusted to 2016 U.S. dollars using U.S. Consumer Price Index [41]. CVD: cardiovascular diseases. * For the standard error we assumed a 25% coefficient of variation.

**Table 2:** Expected lifetime costs and QALYs for an average person exposed to noise associated with the limited use as well as the year-round use of the TNNIS Climb at La Guardia Airport (LGA) in New York.

Scenario	Costs	Incremental Costs	QALYs	Incremental QALYs	ICER ($/QALY)
Limited use of TNNIS	656,173	11,288	18.72	1.13	10,006
Year-round use of TNNIS	644,885		17.6		Reference

QALYs: quality-adjusted life year, ICER: Incremental cost-effectiveness ratio.

**Table 3:** One-way sensitivity analyses for cost-effectiveness of the limited versus the year-round use of TNNIS Climb at La Guardia Airport (LGA) in New York City.

Input Parameters	Low	High
RR of anxiety for aircraft noise exposure (1.00–3.06)	−3574	208,108
Probability of anxiety for people without CVD (0.08 to 0.28)	1694	33,826
Incremental operating costs per person exposed to noise (limited vs. year-round use of TNNIS) (1137 to 1895)	2345	17,668
Health utility decrement for anxiety (−0.26 to −0.06)	6508	21,641
RR of CVD for aircraft noise exposure (1.0–1.18)	5065	15,876
Direct costs of anxiety (2111 to 3518)	6947	13,062
Direct costs of CVD (17,422 to 29,036)	7041	12,972
RR of anxiety for CVD patients (1.49–1.82)	8303	12,109
Prevalence of CVD in general population (0.25 to 0.45)	8431	11,566
Age at baseline (31 to 51)	8625	11,655
Incremental productivity loss per person exposed to noise (limited vs. year-round use of TNNIS) (178.5, 297.5)	8804	11,209
Indirect costs of CVD (9628 to 16,046)	8951	11,062
Health utility decrement for CVD (−0.38 to −0.18)	9374	10,771
Health utility of people with a prior history of CVD (0.74 to 0.94)	9669	10,401
Indirect costs of anxiety (235 to 391)	9668	10,346
RR of CVD for people with a prior history of CVD (1.7 to 2.3)	9764	10,238
Discount rate (0.00 to 0.05)	9978	10,365

CVD: cardiovascular diseases; RR: relative rate; QALYs: quality-adjusted life year, ICER: Incremental cost-effectiveness ratio.

## Supplementary Materials

The following are available online at http://www.mdpi.com/1660-4601/15/8/1753/s1, Figure S1: Number of persons in each census tract within the 60 dB DNL noise contour. dB DNL: dB averaged for a 24-h “day and night” period., Figure S2: Number of persons in each census tract within the 65 dB DNL noise contour. dB DNL: dB averaged for a 24-h “day and night” period.
