# Supplementary material for: The Trade-Off between Optimizing Flight Patterns and Human Health: A Case Study of Aircraft Noise in Queens, NY, USA
Source: Int J Environ Res Public Health. 2018 Aug 15;15(8):1753. doi: 10.3390/ijerph15081753 (PMC6121545; doi:10.3390/ijerph15081753)
Supplement: Supplementary file 1 [file ijerph-15-01753-s001.pdf]

Online Technical Appendix

The trade-off between aircraft flight efficiency and human health: a case study of aircraft noise in Queens, NY

Authors: Zafar Zafari, M.Sc., PhD[1*]; Boshen Jiao MPH[1]; Brian Will, B.Sc.[2]; Shukai Li, B.Sc.[1]; Peter Alexander Muennig, MD, MPH[1]

Affiliations:
1. Health Policy and Management, Mailman School of Public Health, Columbia University, USA
2. Queens Quiet Skies, PO Box 604888, Bayside, NY 11360-4888, USA

Online Appendix 1: The trajectories of the TNNIS and the Whitestone Climbs at LaGuardia Airport (LGA)

Figure S1: The trajectory of the Whitestone Climb at LaGuardia Airport (LGA)
Figure S2: The trajectory of the TNNIS Climb at LaGuardia Airport (LGA)

Online Appendix 2: Population estimate of persons of Community Boards 7 and 11 of Queens, NY, within the 60 and 65 db DNL noise contour under the TNNIS climb

Figure S3: Number of persons in each census tract within the 60 db DNL noise contour
Figure S4: Number of persons in each census tract within the 65 db DNL noise contour

Online Appendix 1: The trajectories of the TNNIS and the Whitestone Climbs at LaGuardia Airport (LGA)

Figure S1. The trajectories of the TNNIS and the Whitestone Climbs at LaGuardia Airport (LGA)

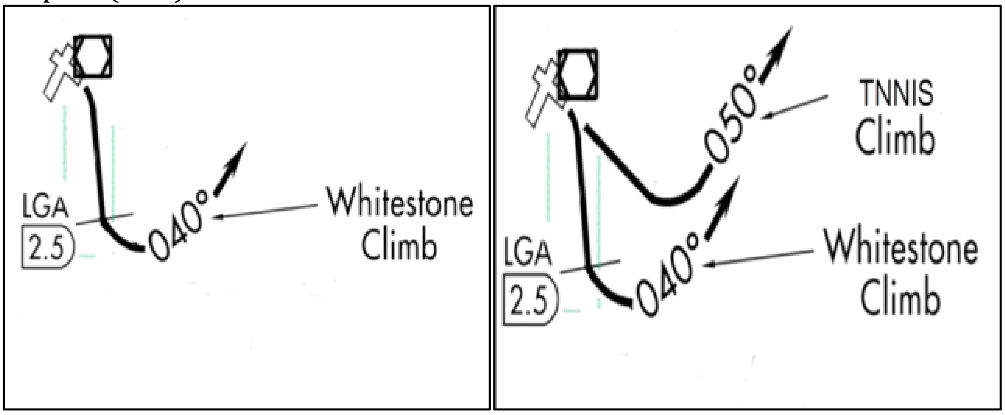

This figure is sourced from the Federal Aviation Administration [1].

Appendix 2: Population estimate of persons of Community Boards 7 and 11 of Queens, NY, within the 60 and 65 dB DNL noise contour under the TNNIS Climb

The estimates for the 60 and 65 dB DNL noise contours were derived from the Port Authority of New York and New Jersey [2]. In addition, the estimates of population were taken from individual census tracts tabulated for the 2010 federal census [3]. We used the map of 60 and 65 dB DNL noise contours to overlay onto a census tract map of northeast Queens, NY. Census tracts outside of the most recently published 60 and 65 dB DNL were not included. In addition, because the focus of this paper was TNNS Climb, census tracts that could have been influenced by other major departure routes such as the Whitestone Climb were not included. Using this method, we were able to estimate the population of census tracts within the 60 and 65 dB DNL noise contours as 83,807, and 31,329, respectively. *Figures S2* and *S3* show the details of number of persons in different census tracts affected by noise contours 60, and 65 dB DNL, respectively.

Figure S2. Number of persons in each census tract within the 60 dB DNL noise contour

Census Tract	# Of Persons
919	5,766
907	1,355
383.01	0
869	2,131
865	4,514
871	1,752
849	7,642
853	5,764
855	6,649
863	7,277
857	5,953
861	2,370
1189	2,468
1201	1,668
859	5,873
797.01	7,055
797.02	4,502
799	3,425
803.01	4,382
803.02	3,504
793	1
383.02	56

Total population of census tracts within 60DNL: 83,807 persons

dB DNL: dB averaged for a 24-hour 'day and night' period.

Figure S3. Number of persons in each census tract within the 65 dB DNL noise contour

Census Tract	# of Persons	Census Tract	# of Persons	Census Tract	# of Persons	Census Tract	# of Persons	Census Tract	# of Persons
Tract # 317, Block # 1008	416	Tract # 357, Block # 1002	141	Tract # 327, Block # 1002	381	Tract # 289, Block # 1001	137	Tract # 871, Block # 1000	21
Tract # 317, Block # 2000	230	Tract # 357, Block # 2003	220	Tract # 327, Block # 1001	473	Tract # 289, Block # 1003	708	Tract # 853, Block # 2000	262
Tract # 317, Block # 3001	126	Tract # 329, Block # 1005	118	Tract # 309.03, Block # 3001	463	Tract # 291, Block # 2000	637	Tract # 853, Block # 1002	675
Tract # 309.04, Block # 1003	106	Tract # 329, Block # 1006	115	Tract # 309.03, Block # 3002	279	Tract # 291, Block # 2001	429	Tract # 853, Block # 1001	298
Tract # 309.04, Block # 1002	53	Tract # 329, Block # 3006	98	Tract # 309.03, Block # 3003	186	Tract # 291, Block # 2002	506	Tract # 871, Block # 1002	187
Tract # 329, Block # 3001	20	Tract # 329, Block # 3007	163	Tract # 309.03, Block # 3004	226	Tract # 291, Block # 2003	457	Tract # 871, Block # 2004	9
Tract # 329, Block # 3000	4	Tract # 329, Block # 3003	179	Tract # 309.03, Block # 3005	201	Tract # 291, Block # 2004	0	Tract # 853, Block # 1000	0
Tract # 329, Block # 1007	68	Tract # 309.04, Block # 1010	276	Tract # 309.03, Block # 3006	246	Tract # 293, Block # 1007	0	Tract # 871, Block # 2011	0
Tract # 329, Block # 3005	0	Tract # 309.04, Block # 1011	131	Tract # 309.02, Block # 3000	404	Tract # 293, Block # 1006	215	Tract # 871, Block # 2009	20
Tract # 329, Block # 1001	0	Tract # 309.04, Block # 1008	30	Tract # 309.02, Block # 3001	13	Tract # 293, Block # 1005	317	Tract # 871, Block # 2000	7
Tract # 329, Block # 1002	163	Tract # 309.04, Block # 1012	78	Tract # 309.02, Block # 4003	364	Tract # 291, Block # 4000	462	Tract # 871, Block # 2007	207
Tract # 329, Block # 1003	207	Tract # 309.04, Block # 2005	303	Tract # 309.02, Block # 4002	467	Tract # 291, Block # 4004	0	Tract # 871, Block # 2002	170
Tract # 329, Block # 1000	225	Tract # 309.04, Block # 2004	397	Tract # 309.02, Block # 4001	268	Tract # 293, Block # 1008	0	Tract # 869, Block # 1014	72
Tract # 347, Block # 2005	105	Tract # 309.04, Block # 2003	110	Tract # 309.02, Block # 4000	331	Tract # 293, Block # 1009	127	Tract # 869, Block # 1009	56
Tract # 329, Block # 2001	229	Tract # 309.04, Block # 2002	12	Tract # 309.03, Block # 1006	408	Tract # 293, Block # 1010	0	Tract # 869, Block #1004	173
Tract # 329, Block # 2000	305	Tract # 309.04, Block # 2001	361	Tract # 309.03, Block # 1005	269	Tract # 261, Block # 1002	335	Tract # 869, Block # 1005	20
Tract # 347, Block # 2011	353	Tract # 309.04, Block # 2000	373	Tract # 309.03, Block # 1004	372	Tract # 261, Block # 1001	180	Tract # 869, Block # 2014	111
Tract # 347, Block # 1002	278	Tract # 329, Block # 3010	321	Tract # 309.03, Block# 1003	306	Tract # 261, Block # 1000	716	Tract # 907, Block # 1026	3
Tract # 347, Block # 2007	199	Tract # 329, Block # 3008	195	Tract # 293, Block # 1000	0	Tract # 263, Block # 1001	36	Tract # 907, Block # 1025	35
Tract # 347, Block # 2008	249	Tract # 329, Block # 3009	0	Tract # 291, Block # 1005	0	Tract # 261, Block # 2000	693	Tract # 919, Block # 1006	536
Tract # 347, Block # 2010	320	Tract # 309.03, Block # 2005	289	Tract # 291, Block # 1004	76	Tract # 261, Block # 3001	800	Tract # 919, Block # 1007	51
Tract # 357, Block # 1001	0	Tract # 309.03, Block # 2004	33	Tract # 291, Block # 1003	247	Tract # 261, Block # 3000	1,280	Tract # 919, Block # 1008	33
Tract # 347, Block # 2009	270	Tract # 309.03, Block # 2003	279	Tract # 291, Block # 1002	364	Tract # 261, Block # 4004	230	Tract # 919, Block # 1004	163
Tract # 357, Block # 1009	9	Tract # 309.03, Block # 2002	393	Tract # 291, Block # 1001	404	Tract # 261, Block # 4003	297	Tract # 919, Block # 1005	196
Tract # 347, Block # 1001	247	Tract # 309.03, Block # 2001	332	Tract # 291, Block # 1000	330	Tract # 871, Block # 1003 (SkyView Parc)	748	Tract # 919, Block # 1003	309
Tract # 357, Block # 1008	285	Tract # 309.03, Block # 2000	381	Tract # 289, Block # 1002	288	Tract # 871, Block # 1001 (City Housing	1,071	Tract # 919, Block # 3012	182

Total population of census tracts within 65 db DNL: 31,329

db DNL: dB averaged for a 24-hour 'day and night' period.

References

1 Federal Aviation Administration. LaGuardia five departure. http://aeronav.faa.gov/d-tpp/1807/00289LAGUARDIA.PDF (accessed 2 Jul 2018).

2 Port authority NY/NJ noise contour map for LaGuardia airport (official noise exposure results of the 14 CFR Part 150 study), meeting presentation for June 21st, 2016. http://panynjpart150.com/LGA_TAC.asp (accessed 1 Mar 2018).

3 2010 New York City population by census tracts. NYC Open Data. https://data.cityofnewyork.us/City-Government/2010-NYC-Population-by-Census-Tracts/si4q-zuzm (accessed 1 Mar 2018).
